# A Comparative Evaluation of Sustainable Binders for Environmentally Friendly Carbon-Based Supercapacitors

**DOI:** 10.3390/nano12010046

**Published:** 2021-12-24

**Authors:** Giovanni Landi, Luca La Notte, Alessandro Lorenzo Palma, Andrea Sorrentino, Maria Grazia Maglione, Giovanni Puglisi

**Affiliations:** 1 Casaccia Research Center, ENEA, Via Anguillarese 301, 00123 Rome, Italy; luca.lanotte@enea.it (L.L.N.); alessandrolorenzo.palma@enea.it (A.L.P.); giovanni.puglisi@enea.it (G.P.); 2 Portici Research Center, ENEA, Piazzale Enrico Fermi 1, 80055 Naples, Italy; mariagrazia.maglione@enea.it; 3 Institute for Polymers, Composites, and Biomaterials—National Research Council (IPCB—CNR), SS Napoli/Portici, Piazzale Enrico Fermi 1, 80055 Naples, Italy; andrea.sorrentino@cnr.it

**Keywords:** water processable, sustainable binder, gelatin, carbon-based supercapacitor, pseudocapacitive material, charge storage mechanisms, faradaic process, cycle stability, aging

## Abstract

Environmentally friendly energy storage devices have been fabricated by using functional materials obtained from completely renewable resources. Gelatin, chitosan, casein, guar gum and carboxymethyl cellulose have been investigated as sustainable and low-cost binders within the electrode active material of water-processable symmetric carbon-based supercapacitors. Such binders are selected from natural-derived materials and industrial by-products to obtain economic and environmental benefits. The electrochemical properties of the devices based on the different binders are compared by using cyclic voltammetry, galvanostatic charge/discharge curves and impedance spectroscopy. The fabricated supercapacitors exhibit series resistance lower than a few ohms and values of the specific capacitance ranged between 30 F/g and 80 F/g. The most performant device can deliver ca. 3.6 Wh/kg of energy at a high power density of 3925 W/kg. Gelatin, casein and carboxymethyl cellulose-based devices have shown device stability up to 1000 cycles. Detailed analysis on the charge storage mechanisms (e.g., involving faradaic and non-faradaic processes) at the electrode/electrolyte interface reveals a pseudocapacitance behavior within the supercapacitors. A clear correlation between the electrochemical performances (e.g., cycle stability, capacitance retention, series resistance value, coulombic efficiency) ageing phenomena and charge storage mechanisms within the porous carbon-based electrode have been discussed.

## 1. Introduction

The rapid development and diffusion of electronic products in everyday life within a broad application range imply an ever-increasing energy consumption. In this context, the opportunity to store energy to deliver power on demand has become a crucial aspect for the more efficient use of energy [1]. Among electrochemical energy storage devices, supercapacitors, also known as electrochemical capacitors, are under intensive academic and industrial investigations in recent years since they have distinct advantages such as higher power density due to the fast charging/discharging rate and long-life stability when compared to batteries and fuel cells [2]. Supercapacitors (SCs) are composed of a medium sandwiched between two high-surface-area electrodes, thus a simple and highly versatile structure that match the requirements for consumer electronics addressed to be smaller, lighter, thinner, and flexible in the near future. Unfortunately, the growing demand for consumer electronics led to large amounts of electrical and electronic waste equipment that pose environmental concerns [3]. Hence, the need for environmentally friendly next-generation smart electronics is urgent. Carbon-based supercapacitors offer the advantage of being fabricated from non-hazardous materials. Residual biomasses such as agriculture and forestry residues and industrial by-products can be used as precursors of the activated carbons adopted for the active material of electrodes [4,5]. Since such materials are in powder form, it is necessary to add a binder to agglomerate the powder particles and produce a slurry. Furthermore, the binder promotes the adhesion between the slurry and the current collector and provides mechanical strength to the electrode. Poly(vinylidene difluoride) (PVDF) is the most widely used binder, as it ensures good electrochemical stability and optimal performances [6]. However, it is processed by using a harmful organic solvent, N-methyl-2-pyrrolidone (NMP) [7], which is also expensive and has a boiling point of about 203 °C, thus requiring high temperatures for its removal during electrode fabrication [8]. Another common polymeric binder is polytetrafluoroethylene (PTFE) that is processable as a dispersion in water but it is a fluorinated material as PVDF [9]. Therefore, research has been focused to introduce sustainable binders in terms of processability, chemical composition, large availability, and facile synthesis. Polyvinyl acetate (PVA) and polyvinylpyrrolidone (PVP) have demonstrated comparable electrochemical performances [10,11]. Research has been focused on natural and naturally derived polymers from renewable resources as well. In the last decade low-cost energy storage devices, sensors and electronics have been developed with this purpose [12,13,14]. Cellulose and its derivatives such as carboxymethylcellulose (CMC) have been extensively applied as a green, abundant and cheap binder in supercapacitors and lithium-ion batteries [15,16]. Other examples range from chitosan [17] and sodium alginate [18], over starch [19] and guar gum [20] to tragacanth gum [21], agarose [22] and casein [23]. Carbonaceous fillers such as activated carbon, graphene or its down-grade form as reduced graphene oxide have been used for the fabrication of electrodes for supercapacitors in sandwich and planar structures [24,25].

In the present study, environmentally benign binders have been used in the active material formulations for the electrode to fabricate water-processable symmetric carbon-based supercapacitors with sodium chloride (NaCl) as an aqueous electrolyte. In particular, three polysaccharides (CMC, chitosan, and guar gum) and two protein-based products (casein and gelatin) have been employed. It is worth noting that gelatin, a renewable animal derivative, has been investigated as a binder for supercapacitors for the first time. The related electrochemical properties have been compared in terms of cycle voltammetry, galvanostatic charge-discharge, and impedance spectroscopy measurements. To evidence the pseudocapacitive behavior of the carbon-based supercapacitors, a detailed analysis regarding the charge storage mechanisms (e.g., surface and diffusion-limited processes) and their combination that contributes to the dielectric properties have been discussed. Additionally, cycle stability and dielectric properties of the fabricated devices after 1000 cycles have been compared to highlight the effect of the aging process at the electrode/electrolyte interface.

## 2. Materials and Methods

### 2.1. Material Preparation

Polyethylene terephthalate (PET) foil (Melinex ST 504, DuPont Teijin Films, Chester, VA, USA, thickness 125 μm) covered with copper (Cu) tape (Kohree, City of Industry, CA, USA, thickness 40 μm) was used as substrate. Henkel Electrodag PF407C graphite ink was deposited on the substrate using a blade coater (Proceq ZAA 2300, Zehntner GmbH Testing Instruments, Sissach, Switzerland) followed by thermal annealing at 90 °C for 30 min, resulting in films with a thickness of about 50 µm. The active material of the electrode was prepared by dissolving activated carbon (Kuraray YP-80F, Tokyo, Japan) obtained from coconut shells and binders in ultrapure water (Milli-Q). The electrode material was prepared according to the following composition: 95 wt.% activated carbon (Kuraray YP 80F, Tokyo, Japan, with characteristic V_micro_ < 2 nm = 0.652 cm^3^/g and specific surface area (SSA) = 2093 m^2^ g^−1^) and 5 wt.% of binder. The investigated binders were CMC (Thermo Fisher, carboxymethyl cellulose sodium salt, Waltham, MA, USA), chitosan (Sigma-Aldrich, chitosan from shrimp shells, Saint Louis, MO, USA), casein (TCI, casein sodium from milk), guar gum (Sigma Aldrich, guar) and gelatin (Sigma-Aldrich, gelatin from porcine skin gel). These biomaterials are water soluble, however, the chitosan needs an acidic solution, therefore acetic acid was added to the formulation. Moreover, the gelatin blend was prepared by adding glycerol to improve its mechanical properties. The gelatin powder was dissolved in a mixture of water and glycerol at 80 °C to obtain a gelatin solution with a concentration of 4.75 wt.%. For more information about the fabrication process, the reader can refer to [24]. The carbonaceous slurry was deposited by blade coating on the PET-Cu-Graphite stack and dried at room temperature. The electrodes were then weighed to obtain the AC mass; the mass loading values were found to range from 3 and 5 mg/cm^2^. In total, 16 samples were prepared for each binder. The electrodes were sorted according to the *m_a_* mass and similar-mass electrodes were paired up for the supercapacitor devices. The electrolyte consisted of an aqueous 1 M NaCl solution, which was drop cast onto a separator made of 40 μm thick Dreamweaver Silver AR40 cellulose paper. The separator was soaked with 250 µL for 5 min. The supercapacitor was completed by facing another electrode to form a sandwich structure. The electrode area was 2.5 × 4 cm^2^. 

### 2.2. Morphology Characterization

The morphology of the sample surface was investigated with a scanning electron microscope (FE-SEM), FEI Phenom desktop scanning electron microscope (Eindhoven, The Netherlands). The high conductive nature of the graphite-based material has allowed samples observation without any preparation, except for the electrical grounding of the surface.

### 2.3. Electrochemical Measurements

The electrochemical characterizations such as cyclovoltammetry (CV), galvanostatic charge-discharge (GCD), and electrochemical impedance spectroscopy (EIS) of the supercapacitors were done on a commercial platform (Arkeo-Cicci Research S.r.l.) at room temperature. The devices were measured in a two-electrode geometry with an average area of about 10 cm^2^. The EIS measurements were performed in the frequency range between 100 mHz and 10 kHz with an ac-signal amplitude of 50 mV at open-circuit voltage. The specific capacitance $C_{S}$ (F/g) of the symmetric SC was computed by integrating the area under the CV curves according to the following equation [26]

(1) $$C_{S}=\frac{2\cdot C_{cell}}{m_{a}}$$

where $C_{cell}=\frac{1}{2\cdot \nu \cdot \left(V_{b}-V_{a}\right)}\cdot \int_{V_{a}}^{V_{b}}i\left(V\right)dV$ is the capacitance of device investigated and $m_{a}$ is the mass of the electrode and it is the sum of the masses of the binder (5%) and activated carbon (95%), respectively. Here, *υ* is the scan rate, *i(V)* is the charging/discharging current and *V_b_* − *V_a_* is the potential window. From the GCD profiles, the equivalent series resistance (*ESR*) can be estimated by

(2) $$ESR=\frac{IR_{drop}}{2\cdot I_{D}}$$

where *IR_drop_* is the voltage drop between the first two points of the discharge plot and *I_D_* is the discharge current. Energy *E* (Wh/kg) and the power *P* (W/kg) densities of the supercapacitors were computed by taking into account the equations:

(3) $$E=\frac{1}{2}\cdot C_{S}\cdot {\left(\Delta V\right)}^{2}=\frac{1}{2}\cdot C_{S}\cdot \frac{{\left(V_{max}-V_{min}-IR_{drop}\right)}^{2}}{3.6}$$

and

(4) $$P=\frac{E}{t_{disc}}\cdot 3600$$

where *V_max_* is the maximum voltage applied to the device, *V_min_* is 0.1 V and *t_disc_* is the corresponding discharge period, respectively.

## 3. Results

**Figure 1:**
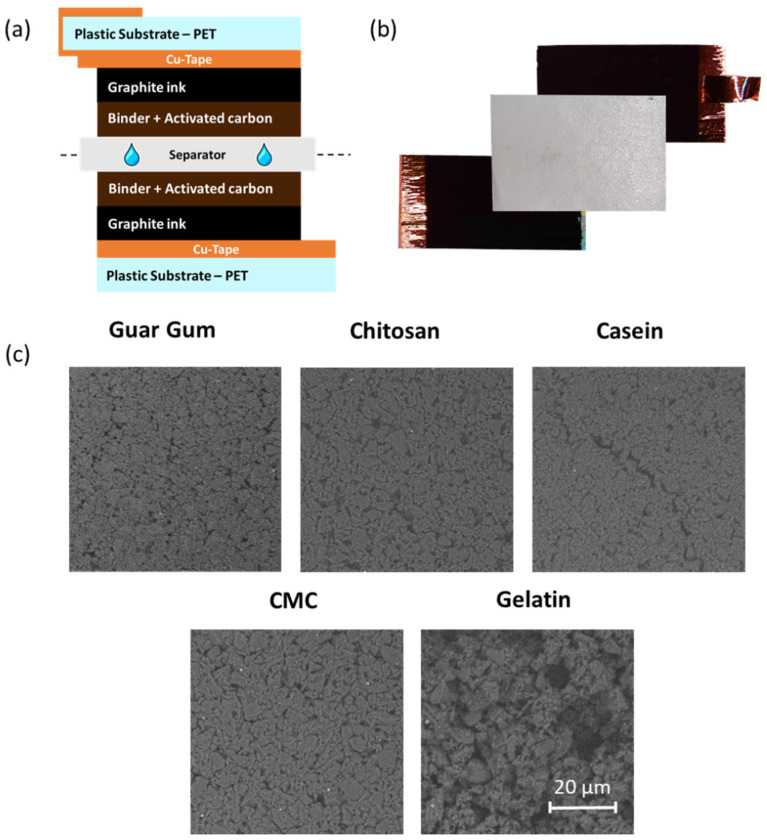
(**a**) Cross-section of the symmetric carbon-based supercapacitors fabricated; (**b**) Photograph of the fabricated device before the assembling; (**c**) SEM images of the surface morphology for the guar gum, chitosan, CMC, casein, and gelatin-based supercapacitors, respectively.

In order to evaluate the dielectric properties of the biomaterials, as alternative and environmentally friendly binders, within low-cost carbon-based supercapacitors a test structure has been used. Figure 1a shows a cross-section of the device formed by a symmetric sandwich structured following the layers sequence: PET/Cu-Tape/Graphite ink/Active material/separator. Moreover, to guarantee the safety of the final device and a low environmental impact, a 1 M NaCl electrolyte has been used. The thickness of the active layer based on AC and biopolymer as a binder has a thickness between 60 and 80 µm, depending on the binder types. In Figure 1b, the image of the components (electrodes and separator) of the device before the assembling are shown. Figure 1c shows the SEM images of the active materials, including the different binders, deposited on the PET/Cu-Tape/Graphite stack. All the samples display relatively homogeneous and dense surfaces without significant holes or cracks; the well-dispersed formulations and the room temperature drying have facilitated the formation of uniform microstructures. Micro cracks are evident only in the case of casein whereas a distinctive morphology is shown when using gelatin due to its gelling properties.

### 3.1. Electrochemical Characterization

**Figure 2:**
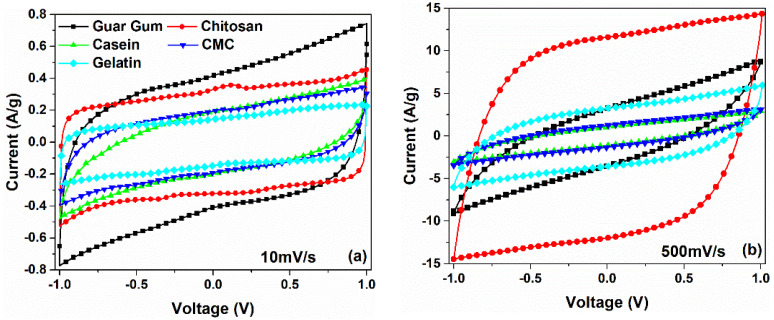
Cycle voltammetry curves of symmetric carbon-based supercapacitors investigated in 1 M NaCl electrolytes measured at (**a**) 10 mV/s and (**b**) 500 mV/s, respectively.

The CV curves of symmetric carbon-based supercapacitors fabricated with different binders (such as guar gum, chitosan, casein, CMC, and gelatin) are shown in Figure 2. To avoid any chemical reactions due to the decomposition of the water within the aqueous electrolyte, the bias voltage has been limited to a range of ±1 V [27]. All the experimental data related to the CV curves, measured with a scan rate from 10 mV/s to 500 mV/s for the devices under investigation, are shown in Figures S1–S5. As can be observed from Figure 2a,b, the investigated devices exhibit a fairly rectangular shape of the voltammetric curves for low (10 mV/s) and high (500 mV/s) sweeping rates, respectively. This shape, without the presence of redox peaks, is a clear indication of the formation of a double-layer capacitance at the interface between electrode and electrolyte [28]. A slightly slanted behavior in the CV curves observed at 500 mV/s suggests the presence of a non-negligible ohmic contribution of the parallel resistance caused by finite conduction through the electrolyte. Moreover, the disappearance of the rectangular shape at 500 mV/s implies the presence of a few ohms of series resistance arising from the ohmic contribution of the carbon electrode [29]. These features can be observed for all the devices investigated, except for the chitosan and the gelatin-based ones, where the dielectric properties of the system are still present even at higher scan rate values.

**Figure 3:**
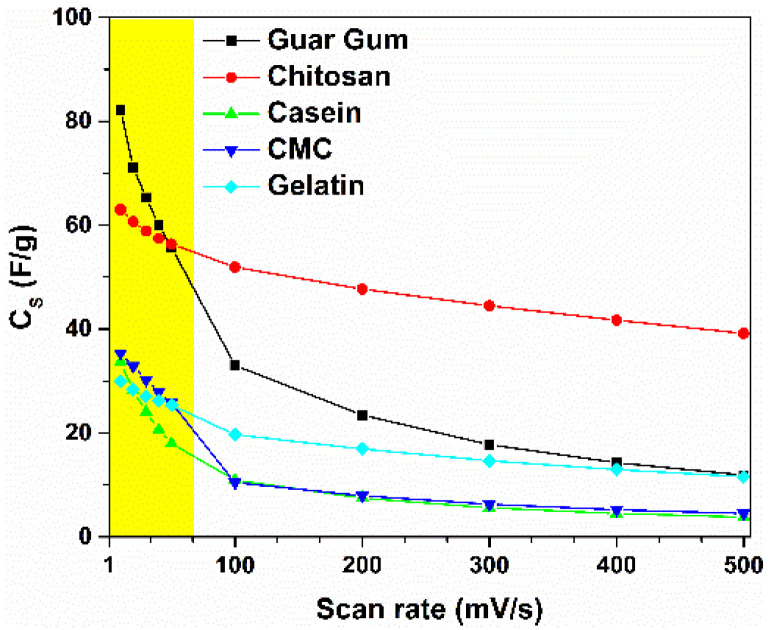
The gravimetric capacitance of the electrode binders investigated as a function of the scan rate.

By taking into account Equation (1), the gravimetric capacitance (*C_S_*) can be calculated from the CV curves. Figure 3 displays the values of *C_S_* as a function of the voltage scan rates, which range from 10 to 500 mV s^−1^. Here, the capacitance values decrease with the increase of the scan rate. At a lower scan rate region (υ ≤ 50 mV/s) the ions have sufficient time to diffuse into the pores of activated carbon at the electrode/electrolyte interface, leading to their accumulation. This phenomenon leads to the formation of a double layer charged at the electrodes, characterized by a capacitance *C_S_*. As can be observed in Figure 3, the highest value of the *C_S_*, which is 82.2 F/g at 10 mV/s, is obtained for *C_S_* having the guar gum as a binder in the electrodes. By increasing the scan rate the *C_S_* value decreases down to 11.9 F/g at 500 mV/s, corresponding to a reduction of 85.6%. Moreover, the device fabricated with chitosan shows a value of 63 F/g at 10 mV/s that decreases only by 37.8%, reaching a stable value of 39.1 F/g at 500 mV/s. The other binders (e.g., casein, CMC, and gelatin) show values of the specific capacitance between 30 and 36 F/g at 10 mV/s that decrease down to 3.8 and 11.6 F/g at 500 mV/s.

It is worth noting that devices fabricated with commercial ACs have a specific capacitance of about 100–150 F/g (depending on the electrolyte in the system) [30]. Lupo et al. report values of *C_S_* for single electrode ranged between 32 and 52 F/g for similar devices fabricated with chitosan as a binder and by using the same aqueous electrolyte 1 M NaCl [17]. By considering the CMC and the casein as binders, the values of *C_S_* reported in the literature ranged between 20–25 F/g, characterized by a percentage weight fraction for the AC of 90% [20,23]. These values are lower than those displayed in Figure 3 in the lower scan rate region. This difference can be related to the different values of the composition fraction and mass loading of the active electrode. In the present study, the value of the wt.% of AC is 95%, whereas the mass loading values are ranged between 3 and 5 mg/cm^2^. To the best of our knowledge, the gelatin has been only used as an alternative binder in the electrodes within the lithium-ion batteries [31]. Moreover, the guar gum has been employed as a binder within the electrode of SC only in combination with other biomaterials such as starch [20]. Therefore, no reference data have been found in the literature for similar device structures for comparison. As can be seen in Figure S6, all the SCs show a marked reduction of *C_S_* value of about 80% for casein, CMC, and guar gum. Conversely, the chitosan and gelatin-based devices exhibit a drop of only 37.8% and 61.4%, respectively. In the latter case, the mechanism involved in the charge storage at the electrodes results to be more efficient compared to the other binders. The large difference between the *C_S_* values computed at lower (slow dynamics) and higher (fast dynamics) scan rate ranges suggests υ-dependent phenomena in the devices. This means that different charge storage mechanisms could take place within the SCs.

### 3.2. Revealing the Charge Storage Mechanisms in the Carbon-Based Supercapacitors

In the energy storage devices (such as supercapacitor/pseudocapacitor and battery) the active material stores charge through a faradaic (electron-transfer via redox reactions) processes, or by the accumulation of ions at an electrical double layer (non-faradaic processes) or by a combination of both [32]. In this latter case, the hybrid characteristics unravel the presence of a pseudocapacitive behavior within the porous carbon-based electrode. In the literature, different methods can be used to estimate and distinguish between these mechanisms by using the electrochemical characterizations (i.e., CV and GCD) toward the calculation of the total charge stored by the electrode material [32,33]. By assuming that the current response *i(V)* of the devices follows a power-law relationship with the sweep rate, the current signal can be written as

(5) $$i\left(V\right)=a\cdot \upsilon^{b}$$

where the constants *a* and *b* are adjustable values depending on potential *V*, sweep rate, and charge storage mechanisms [32]. For a pure capacitive contribution, the exponent *b* is equal to 1 and the current response result to be linearly proportional to scanning rate (i∝ν), which is characteristic of surface-controlled behavior. On the other hand, if the value of *b* is 0.5, the current signal becomes proportional to the square root of the scan rate ($i\propto \upsilon^{0.5}$) due to diffusion-controlled processes. The resulting *i*(*V*) response of the devices is given by the sum of the contributions from the surface-controlled and diffusion-controlled currents and can be written as $i\left(V\right)=i_{diffusive}+i_{capacitive}$ [33]. It should be noted that battery-like electrodes involve diffusion-controlled processes while capacitive and pseudocapacitive electrodes are associated with surface-controlled processes. Values of b ranged between 0.5 and 1 are in a transition area from diffusive-like to capacitive-like responses and, therefore, becomes difficult to discriminate between the two behaviors. However, several authors report that for b values ranging between 0.8 to 1, the surface reactions play a dominant role over diffusion-controlled processes [32,34,35]. As a consequence, the dielectric response of the system is capacitive-like similar to an electrochemical double-layer capacitor (ECDL).

**Figure 4:**
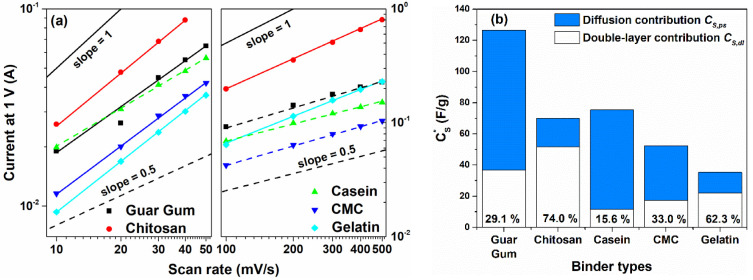
(**a**) Dependence of the current values measured at 1 V for the binders used in the carbon electrode at lower (**left** y-axis) and (**right** y-axis) intermediate scan rate regions, respectively; (**b**) Contribution of pseudocapacitance (diffusion-limited) and double layer capacitance (surface-limited) to the overall capacitance $C_{S}^{*}$ for all the binders investigated. Solid and dashed lines are referred to the capacitive and diffusion-controlled mechanisms, respectively.

Figure 4a shows the current values measured at +1 V for all the binders investigated and for low (*υ* ≤ 50 mV/s) and intermediate (100 ≤ *υ* ≤ 500 mV/s) scan rate ranges, respectively. By using Equation (5), the fitting procedure on the experimental data has been performed. As can be observed, for *υ* ≤ 50 mV/s the resulting *b* values lie in the range between 0.8 and 0.9 except for the casein-based supercapacitor where the exponent shows a value of 0.65. Moreover, at higher scan rates the best-fitting parameters reveal that *b* values are close to 0.5 for the guar gum, casein and the CMC binders. Only for the chitosan and the gelatin-based devices, the exponent values remain constant at 0.9 and 0.8 in the whole sweeping range investigated, respectively. More in detail, the contribution of the capacitive part is dominant at lower scan rate values whereas by increasing the υ the storage mechanism becomes diffusion controlled with *b* approaching 0.5. Here, the devices fabricated are not battery devices, the exponent values of 0.5 means that at the interface electrode/electrolyte the absorbed ions transfer charge with the electrode coming from de-solvated and adsorbed ions. These faradaic reactions take place on the surface or near-surface of the electrode materials and are characterized by a fast sequence of reversible surface-controlled electrochemical reactions redox or electrosorption processes [36]. These findings suggest that the near-rectangular CV profile, reported in Figure 2, arises from a pseudocapacitance behavior with the occurrence of both of the charge storage mechanisms within the active electrode [37].

Since the device structure, AC amount, and the electrolyte are the same for all the SCs investigated, only the different types of binder could lead to the formation of pseudocapacitance behavior at the electrode. The polymer structure of the binders studied contains many carboxyl, hydroxyl, and amino groups on the backbone that could modify the physical and chemical properties of the carbon surface (e.g., polarity and wettability) [38,39,40]. As reported in the literature, this mechanism causes a beneficial effect on the device performance, generating a pseudocapacitance behavior due to the faraday redox reaction [38]. 

The diffusion-limited contribution $i_{diffusive}$ is $\propto \upsilon^{0.5}$, whereas surface limited contribution $i_{capacitive}$ is ∝ν; therefore, Equation (5) can be written as [41]:

(6) $$i\left(V\right)=k_{1}\nu +k_{2}\upsilon^{0.5}$$

where $k_{1}$ and $k_{2}$ are suitable values. The $k_{1}\nu$ contribution is due to the capacitive part while $k_{2}\upsilon^{0.5}$ is the diffusion-limited part, respectively. To estimate and distinguish between these two contributions Trasatti and Dunn provide a method to calculate the total charge stored by the pseudocapacitive material under study [33,35]. Similarly to Equation (6), the total voltammetric charge *q*(*υ*) could be expressed as a function of scan rate through the following equation [33]

(7) $$q_{S}\left(\upsilon \right)=q_{\infty }+k\upsilon^{-0.5}$$

where $k\upsilon^{-0.5}$ represents charge storage related to semi-infinite diffusion, k is a constant, and $q_{\infty }$ is the charge stored at a high scanning rate (*υ* → ∞). The double-layer charge, $q_{dl}$ (very similar to $q_{\infty }$), can be estimated from the extrapolation of *q_s_* vs. $\upsilon^{-0.5}$ (see Figure S7a). In the partition procedure, the total voltammetric charge, $q_{S}$, can be extracted from the plot of 1/*q_s_* vs. $\upsilon^{0.5}$ (see Figure S7b). Accordingly, the pseudocapacitance charge, *q_ps_*, can be obtained from the difference between $q_{S}$ and $q_{dl}$ [42]. In addition, the quantity $C_{S}^{*}$, $C_{S,dl}$, and $C_{S,ps}$-corresponding to the maximum total specific capacitance at *υ* → 0, double-layer capacitance, and pseudocapacitance-can be obtained by dividing the charge by the potential window of CV (i.e., 2.0 V in this work), which are displayed in Figure 4b. Here, the extracted values obtained by the partition method are in good agreement with what is reported in Figure 3.

Figure 3 and Figure 4a right y-axis suggest that by increasing the sweep rate (*υ* ≥ 100 mV/s) the total charge is stored electrostatically in the double layer (blue filled histograms in Figure 4b) because faradaic reactions are too slow to occur. Furthermore, when the sweep rate approaches zero, the total charge storage can be estimated as the sum of both faradaic and double layer charge storage mechanisms that can occur concurrently at the electrode surfaces. However, only the chitosan and the gelatin report a higher value of the double-layer contribution (at least 60%) to the total capacitance that suggests a merely pure capacitance behavior. This result validates the good retention properties shown in Figure S6. Conversely, the other binders such as guar gum, casein, and CMC show a marked pseudocapacitance behavior with more than 70% of $C_{S}^{*}$ originated by the fast faradaic reactions. This latter mechanism induces better dielectric performances but lower retention values. These findings justify the higher values of *C_S_* reported for υ ≤ 50 mV/s and the capacitance drop at the intermediate (100 ≤ υ ≤ 500 mV/s) scan rate region.

**Figure 5:**
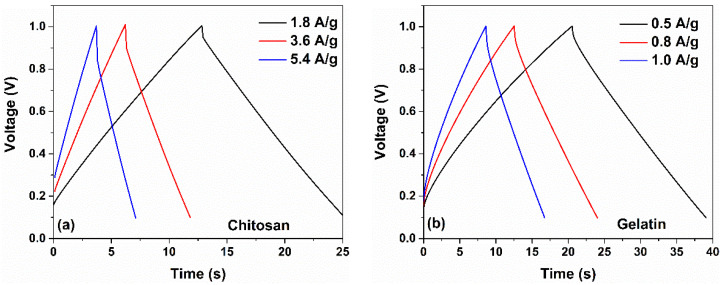
Galvanostatic charge and discharge curves at various current densities for the (**a**) chitosan and (**b**) gelatin-based supercapacitors in 1 M NaCl electrolytes, respectively.

**Figure 6:**
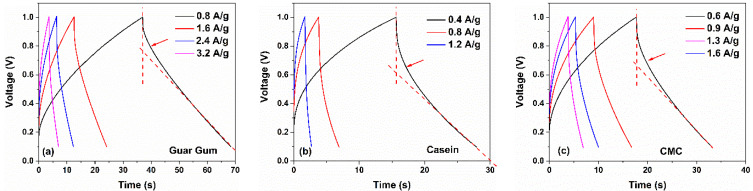
Galvanostatic charge and discharge curves at various current densities for the (**a**) guar gum, (**b**) casein and (**c**) CMC-based supercapacitors in 1 M NaCl electrolytes, respectively.

**Figure 7:**
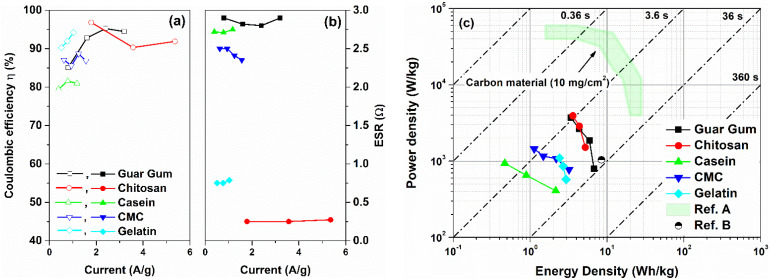
Current dependence of the (**a**) Coulombic efficiency and (**b**) equivalent series resistance values as a function the binders investigated, respectively; (**c**) Ragone plot of gravimetric power density versus gravimetric energy density for the investigated electrodes in comparison with other reported symmetric environmentally friendly aqueous carbon-based SCs with their associated time constant regimes. The references [A] and [B] correspond to [45,46], respectively.

Similar to the CV curves, the presence of a pseudocapacitance contribution modifies the galvanostatic charge-discharge profiles. The GCD curves measured at different current densities for the investigated supercapacitors are displayed in Figure 5 and Figure 6. As displayed in Figure 5, for devices with a dominant capacitive contribution of the double layer (e.g., chitosan and gelatin-based electrodes) linear charging and discharging curves have been observed. On the other hand, devices fabricated with guar gum, casein, and CMC report non-linear GCD curves, manifested as a curvature at the beginning of the discharge profile, as indicated by arrows in Figure 6. This behavior is originated from the faradaic current that comes from the charge redistribution processes in the electrode surface [43]. Further evidence of the pseudocapacitive behavior at the electrode/electrolyte interfaces is revealed in the coulombic efficiency η of the devices. This quantity η can be calculated as the ratio between discharging time and charging time when the charge–discharge current densities are equal. In Figure 7b, the η values computed from the GCD profiles as a function of the current density are shown. As can be observed, the efficiency is lower than 100%, indicating that some contributions to the capacitance *C_S_* arise from the pseudocapacitance [43,44]. Here, the η values are ranged between 80 and 95% for all the binders under investigation.

By considering the IR drop observed in GCD profiles, the ESR value can be estimated. In Figure 7a, the ESR values extracted from Equation (2) as a function of the current densities for all the supercapacitors investigated are shown. Devices characterized by a near-rectangular shape of the CV loop, based on chitosan and gelatin as a binder, reveal lower values of ESR that are 0.25 Ω and 0.75 Ω, respectively. These values are lower than one order of magnitude compared to those reported in the literature for similar binders [17,23]. Furthermore, the other biomaterials (e.g., guar gum, casein, and gelatin) show series resistance values ranging between 2.5 Ω and 3 Ω. These promising values of ESR are related to the low resistance of the electrode, due to a large amount of activated carbon within, and by the use of a 1 M aqueous electrolyte.

Figure 7c shows the Ragone plot of specific power versus specific energy for the devices under test. The diagonal dashed-dotted lines represent the characteristic operation timescales, obtained by dividing the energy by the power (τ=E/P). The values of E and P have been calculated by using Equations (3) and (4), respectively. All the SCs investigated report values in good agreement with what is found in the literature for carbon-based SCs (green area in Figure 7c) [45,46]. Moreover, the resulting operation time τ is ranged between a few seconds to tens of seconds, as expected by the SC applications. Although the green and alternative materials have been investigated in the last decade for energy applications (e.g., in Li-ion batteries and supercapacitors), only a few studies are present in the literature for devices fabricated entirely with sustainable binders and aqueous electrolytes [17,46]. To the best of our knowledge, only chitosan has been studied as a binder for fully eco-friendly supercapacitors [46]. For the other materials (e.g., CMC and casein), to improve the voltage window and, therefore, the energy performance, the literature works report devices where the electrolyte is not environmentally friendly. Moreover, the guar gum has been used only in combination with potato starch [20]. On the other hand, gelatin has been not yet used as a binder in carbon-based supercapacitors. As a consequence, the reference data for these biomaterials are not reported in Figure 7c.

As can be seen, the guar gum and the chitosan-based supercapacitors are the most performant devices. Both the SCs can deliver an average value of energy and power densities of 3.6 Wh/kg and 3925 W/kg, respectively. The gelatin, which is the new material used as a binder within a symmetric carbon-based supercapacitor, shows an average value of energy and power densities of 3.0 Wh/kg and 1000 W/kg, respectively. Here, for all the binders investigated, the operating time ranges between 3.6 s and 36 s depending on the discharging current. However, to enhance the device performance further investigations on the electrode composition (e.g., use of new binders and conductive fillers) and electrolytes (e.g., use of hydrogel) will be carried out.

### 3.3. Analysis of the Cycle Stability and the Ageing Phenomena within the Carbon-Based Supercapacitors

**Figure 8:**
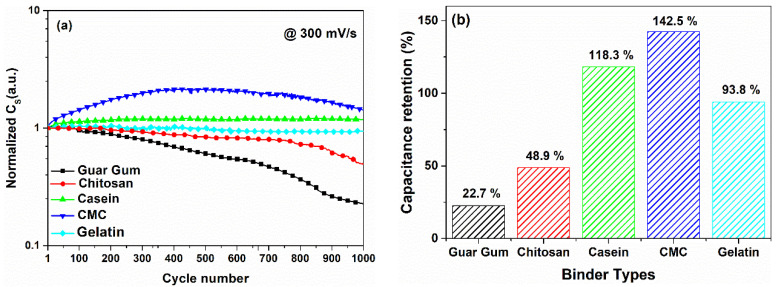
(**a**) Cycles stability of the SCs investigated in 1 M of NaCl performed with 1000 CV cycles at 300 mV/s in the voltage range between 0 and 1 V; (**b**) comparison of the capacitance retention after 1000 cycles at 300 mV/s as a function of the binder types.

Figure 8a shows the endurance of the devices under cycle voltammetry measurements performed at 300 mV/s and in the voltage range ±1. The capacitance values measured for the SCs investigated are in good agreement with what has been reported in Figure 3. As evidenced in Figure 8b, the supercapacitors based on CMC, casein, and gelatin highlight a stable behavior of the dielectric properties up to 1000 working cycles. In addition, the CMC and the casein show an increase in the capacitance value ranging between 20 and 40% at the end of the cycling procedure. This trend has been already observed in the literature for systems where faradaic reactions occur near the electrode surface immersed in electrolyte [47,48]. This phenomenon can be related to the slow insertion of the electrolyte into the bulk structure of the electrode material. Here, the diffusion of ions causes a formation of a greater number of active sites within the electrode material and, therefore, an enhance of the cyclability. Conversely, the chitosan and guar gum-based SCs reveal a monotonic decrease of the *C_S_* values as the cycle number increases. In particular, the decrement for the chitosan is about 50% compared to its initial value; whereas, the decrease for the guar gum is about 80%. For the gelatin and the chitosan-based devices, the dominant charge storage mechanism is surface limited and they work as a double-layer capacitor. However, the cycle stability for both supercapacitors is different. It seems that for the devices investigated, the cycle stability is not directly correlated to the charge storage mechanisms (e.g., surface and diffusion controlled) at the electrode/electrolyte interface but depends only on the biomaterial used as a binder in the electrodes. 

**Figure 9:**
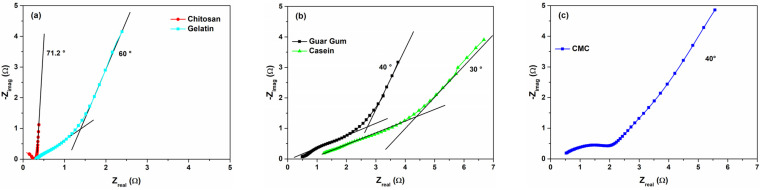
Comparative Nyquist plots for the SCs with (**a**) chitosan, gelatin, (**b**) guar gum, casein, and (**c**) CMC as the electrode material binder in the fresh state.

In order to investigate the interplay between the surface and the diffusion processes that contribute to the overall capacitance value, the impedance spectra for the fabricated devices have been measured. The impedance spectroscopy has been extensively used in electrochemistry and energy applications from generation to storage (e.g., solar cells, batteries, and supercapacitors) [32,49,50]. Figure 9 shows the Nyquist plots—presenting the imaginary part, −Z_imag_, as a function of the real part, Z_real_, of the complex impedance—as a function of the binder types for the fresh state devices. As expected, all the curves have a long tail at lower frequencies that is a typical shape observed for the charge storage mechanisms of the capacitive and pseudocapacitive materials and their associated interfacial phenomena [32]. In the literature, several studies report EIS models that describe porous electrodes in electrolyte solutions [32,50,51,52]. When the porous carbon electrodes physically store charges without any transfer (only charge accumulation) the resulting impedance spectra do not contain the semicircle in the high-frequency region. A supercapacitor can be modeled using a combination of resistive and capacitive elements. In this case, a simple vertical line can be observed in the Nyquist plot. However, for pseudocapacitance behavior where a faradaic reaction occurs at the surface, a second slope of the imaginary part of the impedance in the low-frequency region has been reported [50,51]. It should be noted that when functional groups or dopants at the surface of the carbon materials contribute to charge transfer events or some faradaic reactions occur in pseudocapacitive materials, a semicircle could appear in the spectra [32,52]. 

Devices where the double layer contribution to the overall capacitance is dominant, such as chitosan and gelatin, display a vertical straight line nearly parallel to the imaginary axis (−Z_imag_) at a lower frequency range. Here, the different values of the frequency shift of the imaginary part of the impedance from the theoretical 90° of a pure double-layer capacitance are attributed to the porosity of the carbon-based electrode [32]. As can be noted in Figure 9, the frequency shift is 70° and 60° for the chitosan and gelatin, respectively. Moreover, the lower value of the resistance (<1 Ω) suggests high electrode conductivity and good electron-transfer rates [52]. On the other hand, when the charge transfer processes become less efficient, the resistance increases and the diffusion contribution to the *C_S_* value becomes dominant. 

In Figure 9b,c, the impedance spectra for the CMC, guar gum, and casein are shown. In good agreement with what has been reported in Figure 7b, the devices characterized with a higher value of ESR show experimental spectra shifted towards a higher resistance range (2–4 Ω). This means that a diffusion layer near the electrode interface is present with a non-negligible resistance value [53]. The resulting value of the frequency shift decreases down to 40°, compared to the chitosan reference device, suggesting the presence of a major contribution originated by the diffusion. Depending on the time constant of the electrode kinetics, a semicircle at an intermediate frequency range could appear in the Nyquist plot. Here, only the curve related to the CMC-based supercapacitor shows a clear semicircle loop with a diameter of about 1.9 Ω in the frequency range investigated. This low value means that charge-transfer processes occur at an electrode interface. Moreover, the decreasing of the phase shift down to 40° suggests a diffusion-limited/capacitive response that can be easily described and modeled by a Randles equivalent circuit model [12]. In our devices, the binders contain atoms (such as Na in casein and CMC) and functional groups (e.g., carboxyl, hydroxyl, and amino) that could interact with the activated carbon within the electrolyte. Several authors report interactions between hydroxyl ions and cation species (e.g., Na^+^ through the aqueous electrolyte), that originate redox transitions at the electrode surface [31,44]. For example, carboxyl and hydroxyl groups in CMC can release O content that can lead to a self-doping of the carbon materials [38,39,40,54]. These findings confirm that the pseudocapacitance behavior observed at the electrode interface, for the SCs investigated, is also related to the binder type employed.

**Figure 10:**
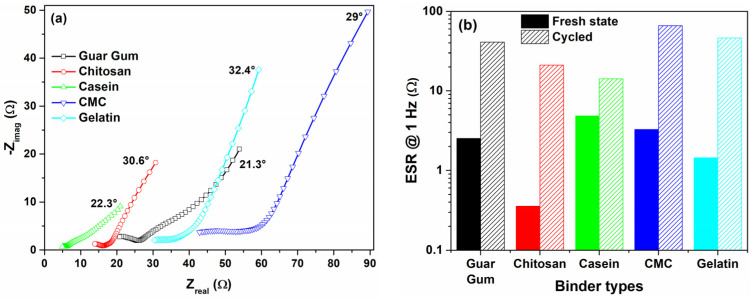
(**a**) Nyquist plots of the supercapacitors investigated after the cycling procedure; (**b**) Comparison of the ESR value at 1 Hz as a function of the binder types extracted from the impedance spectra for the fresh and the cycled devices, respectively.

The effect of the cycling procedure performed on the devices for 1000 cycles has been evaluated by using impedance spectroscopy measurements. In Figure 10a, the impedance spectra measured of the cycled devices for different binders have been reported. Additionally, a comparison of the ESR values estimated from the impedance spectra at 1 Hz between the fresh and cycled SCs is shown in Figure 10b. It is well known that an increase of the resistance after cycling stress suggests that faradaic reactions take place on the electrode surface corresponding to corrosion of the surfaces and/or oxidation of the electrolyte [55]. In our samples, the endurance test after CV cycles has led to an overall increase in electrical resistance. All the devices undergo an accelerated aging process induced by repetitive cycles that leads to a modification of the CV loop. As a result, the devices become more resistive and the impedance spectra shift towards higher resistance. Chitosan and guar gum-based supercapacitors, which are characterized by a prominent capacitance loss, show a marked increase in the ESR values, one and two orders of magnitude compared to the fresh devices, respectively. On the other hand, the casein, CMC, and the gelatin electrodes show a minor change of the capacitance value notwithstanding an increment of the ESR values. The presence of a long tail at a low-frequency region in the Nyquist plots suggests that a pseudocapacitance behavior persists within the aged supercapacitor. Here, a residual phase shift value between 20° and 30°, is still present indicating the existence of light diffusion processes within the devices at lower frequencies.

## 4. Conclusions

Symmetric carbon-based supercapacitors with environmentally friendly binders, such as gelatin, chitosan, casein, guar gum, and carboxymethyl cellulose have been fabricated by using a simple and low-cost water solution process. Among the biomaterials investigated, gelatin is a novel binder used to fabricate electrodes. Aqueous NaCl electrolyte guarantees safety and a low environmental impact of the final devices, compared to flammable and harmful organic electrolytes. The resulting supercapacitors have gravimetric capacitance values between 30 and 80 F/g with few ohms of series resistance and good coulombic efficiency. The large difference between the capacitance values computed at lower (slow dynamics) and higher (fast dynamics) scan rate ranges suggests υ-dependent phenomena in the devices. By using the partition method, a pseudocapacitance behavior has been found at the electrode/electrolyte interface. Different charge storage mechanisms take place within the SCs where the surface and diffusion-limited processes are concurrently at the electrode interface. Gelatin and chitosan-based devices show a capacitive-like dielectric response similar to an electrochemical double-layer capacitor. Conversely, for the CMC, casein, and guar gum-carbon-based electrodes, the diffusion contribution to the overall capacitance result was found to be dominant. The pseudocapacitance behavior also affects the impedance spectra with the presence of a long tail at a low-frequency region in the Nyquist plots. Only the gelatin and the chitosan-based devices show impedance spectra similar to a double-layer capacitor. After an accelerated aging procedure performed on the devices for 1000 voltammetry cycles, the dielectric properties, and the pseudocapacitance behavior change. The devices become more resistive suggesting modifications of the electrode surface due to the irreversible faradaic reactions and/or oxidation of the electrolyte. The lower value of the residual phase shift indicates that the diffusion processes within the cycled devices are still present. An increased capacitance retention rate for the casein and CMC-based devices of 120% and 140% after 1000 cycles have been observed, respectively.

## Data Availability

The data presented in this study are available on request from the corresponding author.

## Supplementary Materials

The following are available online at https://www.mdpi.com/article/10.3390/nano12010046/s1, Figure S1. Cycle voltammetry curves of symmetric carbon-based supercapacitors fabricated with Guar gum as a binder in 1 M NaCl electrolytes measured at (a) lower and (b) intermediate scan rate regions, respectively. Figure S2. Cycle voltammetry curves of symmetric carbon-based supercapacitors fabricated with chitosan as a binder in 1 M NaCl electrolytes measured at (a) lower and (b) intermediate scan rate regions, respectively. Figure S3. Cycle voltammetry curves of symmetric carbon-based supercapacitors fabricated with casein as a binder in 1 M NaCl electrolytes measured at (a) lower and (b) intermediate scan rate regions, respectively. Figure S4. Cycle voltammetry curves of symmetric carbon-based supercapacitors fabricated with CMC as a binder in 1 M NaCl electrolytes measured at (a) lower and (b) intermediate scan rate regions, respectively. Figure S5. Cycle voltammetry curves of symmetric carbon-based supercapacitors fabricated with gelatin as a binder in 1 M NaCl electrolytes measured at (a) lower and (b) intermediate scan rate regions, respectively. Figure S6. Capacitance percentage loss as a function of the binder types. Figure S7. (a) Dependence of *q_S_* on *υ*^−0.5^ and (b) of 1/*q_S_* on *υ*^0.5^ for the binders investigated in 1 M NaCl.
