# Supplementary material for: A Comparative Evaluation of Sustainable Binders for Environmentally Friendly Carbon-Based Supercapacitors
Source: Nanomaterials (Basel). 2021 Dec 24;12(1):46. doi: 10.3390/nano12010046 (PMC8746753; doi:10.3390/nano12010046)
Supplement: Supplementary file 1 [file nanomaterials-12-00046-s001.zip › nanomaterials-1503574-supplementary.pdf]

nanomaterials

Supplementary Materials

A Comparative Evaluation of Sustainable Binders for Environmentally Friendly Carbon-Based Supercapacitors

Giovanni Landi [1,2,*], Luca La Notte [1], Alessandro Lorenzo Palma [1], Andrea Sorrentino [3], Maria Grazia Maglione [2] and Giovanni Puglisi [1]

1 ENEA, Casaccia Research Center, Via Anguillarese 301, 00123 Rome, Italy; luca.lanotte@enea.it (L.L.N.); alessandrolorenzo.palma@enea.it (A.L.P.); giovanni.puglisi@enea.it (G.P.)
2 ENEA, Portici Research Center, Piazzale Enrico Fermi 1, 80055 Naples, Italy; mariagrazia.maglione@enea.it
3 Institute for Polymers, Composites, and Biomaterials—National Research Council (IPCB—CNR), SS Napoli/Portici, Piazzale Enrico Fermi 1, 80055 Naples, Italy; andrea.sorrentino@cnr.it
* Correspondence: giovanni.landi@enea.it; Tel.: +39-0817723627

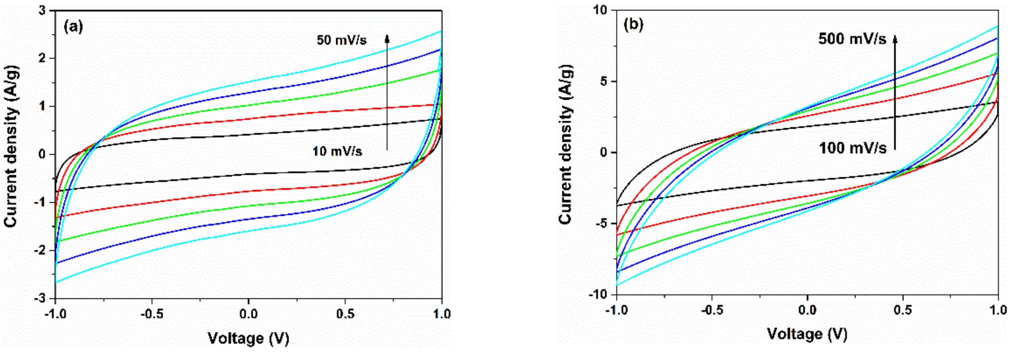

Figure S1. Cycle voltammetry curves of symmetric carbon-based supercapacitors fabricated with Guar gum as a binder in 1 M NaCl electrolytes measured at (**a**) lower and (**b**) intermediate scan rate regions, respectively.

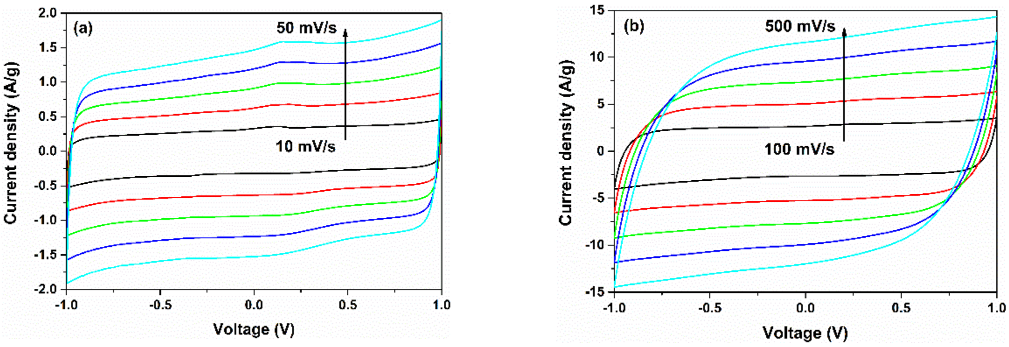

Figure S2. Cycle voltammetry curves of symmetric carbon-based supercapacitors fabricated with chitosan as a binder in 1 M NaCl electrolytes measured at (**a**) lower and (**b**) intermediate scan rate regions, respectively.

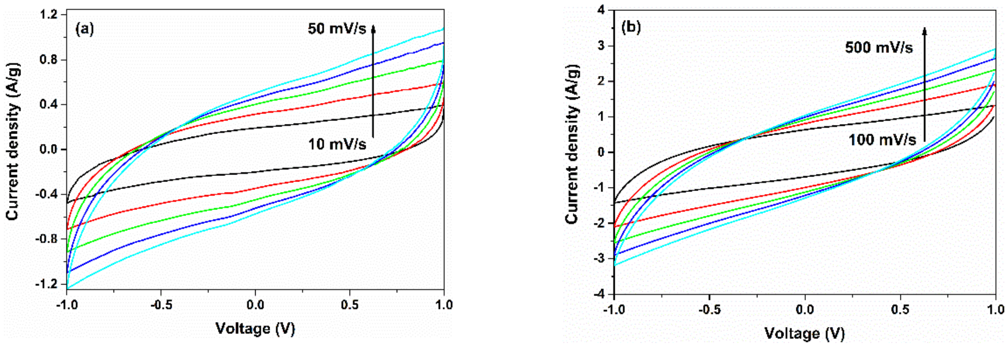

Figure S3. Cycle voltammetry curves of symmetric carbon-based supercapacitors fabricated with casein as a binder in 1 M NaCl electrolytes measured at (**a**) lower and (**b**) intermediate scan rate regions, respectively.

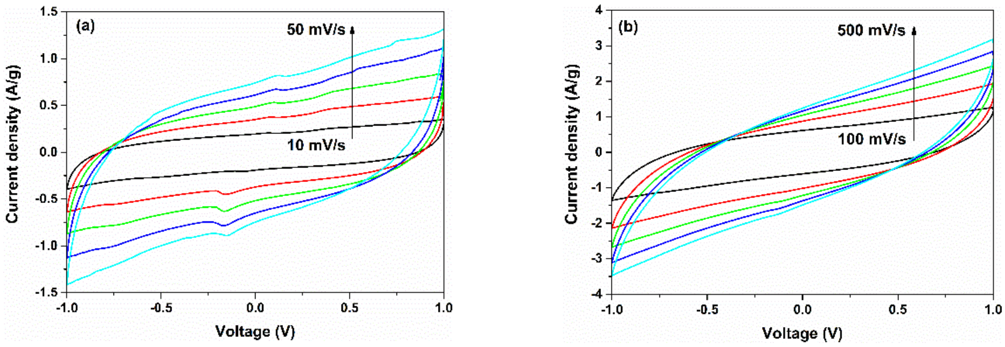

Figure S4. Cycle voltammetry curves of symmetric carbon-based supercapacitors fabricated with CMC as a binder in 1 M NaCl electrolytes measured at (**a**) lower and (**b**) intermediate scan rate regions, respectively.

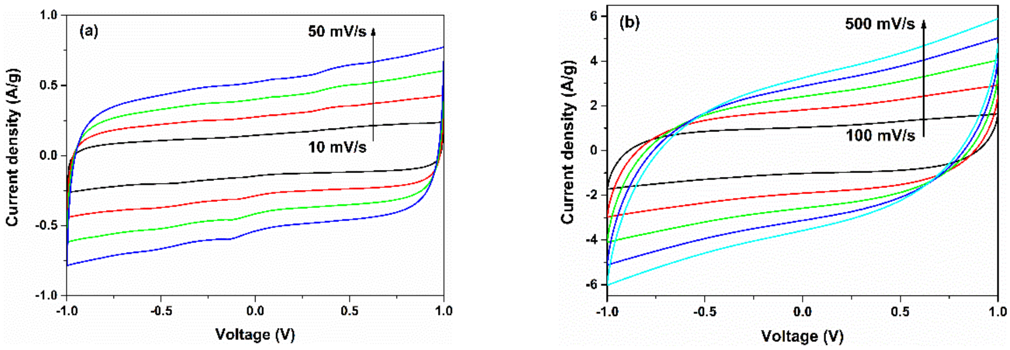

Figure S5. Cycle voltammetry curves of symmetric carbon-based supercapacitors fabricated with gelatin as a binder in 1 M NaCl electrolytes measured at (**a**) lower and (**b**) intermediate scan rate regions, respectively.

In order to quantify the reduction of the capacitance value as a function of the voltage scan rate, observed for all the binders used, the ratio $L = C_{10}/C_{500}$ has been computed. Here, C_{10} and C_{500} correspond to the C_S values at 10 mV/s and 500 mV/s, respectively. Figure S6 shows the percentage loss L of the capacitance C_S as a function of the binder types.

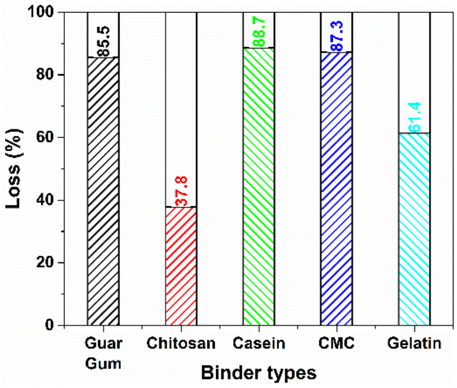

Figure S6. Capacitance percentage loss as a function of the binder types.

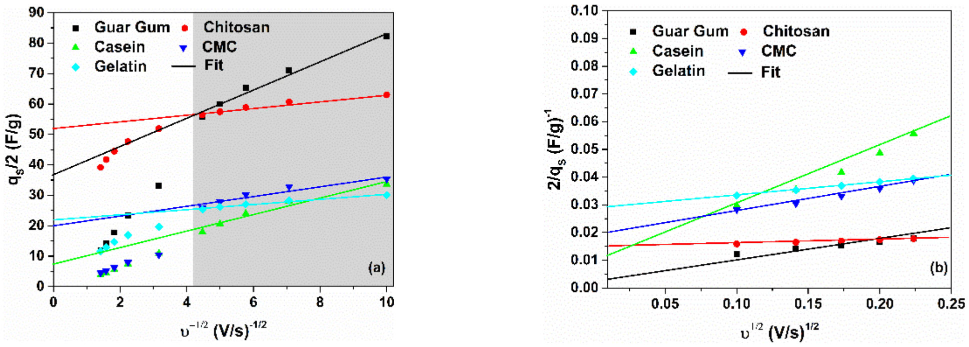

Figure S7. (**a**) Dependence of q_s on $\upsilon^{-0.5}$ and (**b**) of 1/q on $\upsilon^{0.5}$ for the binders investigated in 1 M NaCl.
